# Early Stage Development of a Newcastle Disease Vaccine Candidate in Corn

**DOI:** 10.3389/fvets.2020.00499

**Published:** 2020-08-24

**Authors:** Naila Shahid, Tahir Rehman Samiullah, Sana Shakoor, Ayesha Latif, Aneela Yasmeen, Saira Azam, Ahmad Ali Shahid, Tayyab Husnain, Abdul Qayyum Rao

**Affiliations:** Centre of Excellence in Molecular Biology, University of the Punjab, Lahore, Pakistan

**Keywords:** plant-based vaccine, Newcastle disease virus, infectious diseases, poultry, immunoassay, recombinant protein, antibody, corn

## Abstract

Newcastle disease (ND) is a viral disease that causes labored breathing, periorbital oedema, and ataxia in the majority of avian species. The available vaccines against Newcastle disease virus (NDV) are limited, owing to their low reactivity and multiple dosage requirements. Plant-based machinery provides an attractive and safe system for vaccine production. In the current study, we attempted to express fusion (F) and hemagglutinin-neuraminidase (HN) proteins (the protective antigens against NDV) under constitutive 35S and seed-specific Zein promoters, respectively. Almost 2–7.1-fold higher expression of F gene mRNA in transgenic corn leaves and 8–28-fold higher expression of HN gene mRNA in transgenic corn seeds were observed, when the expression was analyzed by real-time PCR on a relative basis as compared to non-transgenic control plant material (Leaves and seeds). Similarly, 1.66 μg/ml of F protein in corn leaves, i.e., 0.5% of total soluble protein, and 2.4 μg/ml of HN protein in corn seed, i.e., 0.8% of total seed protein, were found when calculated through ELISA. Similar levels of immunological response were generated in chicks immunized through injection of *E. coli*-produced pET F and pET HN protein as in chickens orally fed leaves and seeds of maize with expressed immunogenic protein. Moreover, the detection of anti-NDV antibodies in the sera of chickens that were fed maize with immunogenic protein, and the absence of these antibodies in chickens fed a normal diet, confirmed the specificity of the antibodies generated through feeding, and demonstrated the potential of utilizing plants for producing more vaccine doses, vaccine generation at higher levels and against other infectious diseases.

## Introduction

Poultry is one of the largest groups of livestock worldwide. Newcastle disease is an infectious disease that is contagious, septicaemic, fatal, and destructive to poultry, including to chickens and turkeys (1). The outbreak of this disease has resulted in huge economic losses to the poultry industry worldwide. Newcastle disease also has devastating effects on domestic poultry production (2). The spread of diseases in wild and domestic birds can only be prevented through timely disease diagnosis and mass application of effective vaccines (1, 3). Newly developed plant-based vaccines are efficient, affordable, easy to produce, convenient to store and transport, and appropriate for immunizing a large number of farm animals through feed; thus, plant-based vaccines can prevent economic losses caused by the outbreak of this notorious disease (4–7). Additionally, the development of plant-based vaccines for veterinary diseases is a rapidly emerging topic of interest in plant molecular biology. Many attempts have been made to produce plant-based vaccines that protect against multiple veterinary diseases (7–12).

Traditional vaccines and diagnostic tests are available for the prevention and diagnosis of NDV, but developing new vaccines against NDV is a major concern. Currently used NDV vaccines are traditional vaccines that are becoming outdated. The first clinical trials using live attenuated vaccines began in 1935–1941 (13). Live and inactivated vaccines have been used to prevent the endemic diseases throughout the world (14). However, an advantage of subunit vaccine is that requires only immunogenic portion of target virus and reduces the risk of spread and recombination. The major problems related to live and inactivated vaccines are that propagation and inactivation of viruses are expensive due to strict quality controls and sometime improper inactivation could be a major source of disease outbreaks (15, 16). Although traditional vaccines are still in use, their application often results in the appearance of symptoms associated with the target disease and failure of animals to respond to the vaccine in field conditions. The most frequently used method of vaccination against NDV is intranasal. Intranasal vaccination induces mucosal immunity but always poses some potential risks to the birds. Plant-based vaccines are an alternative approach to inducing mucosal immunity, which is the most important factor for providing protection against viral respiratory diseases (4, 15, 17).

ND is one of the most infectious diseases of poultry caused by NDV (17). The poultry industry plays an important role in the economy of the village, with 3,611 million eggs and 100.42 metric tons of poultry meat generated annually. Newcastle disease's panzootic occurred worldwide, with epizootics specifically reported in Africa, Asia, South and Central America and sporadic epizootics occurring in Europe (18). NDV is an enveloped virus of the *Paramyoxviridae* family (APMV-1), which has a negative single-stranded RNA genome and 15,186 nucleotides. There are 6 transcriptional units in the NDV genome, which is 3′-N–P-M-F–HN-L-5′ (19, 20). Transcription of the HN unit and the F unit is responsible for the constitution of the HN (hemagglutinin-neuraminidase) and F (fusion) protein, respectively. On the envelope of the virus, both of these proteins act as virus neutralizing and protective antigens of NDV (1, 21, 22). The F protein of NDV is basically a type I membrane glycoprotein. The F protein is produced as an inactive precursor, F_0_, and the cleavage of this precursor into two subunits is essential for viral entry and cell-to-cell fusion. The cleavage site is well-characterized and is considered a major determinant of NDV pathogenicity in chickens. After activation, a series of structural changes occur in the F protein, resulting in the fusion of the envelop and membrane, which leads to membrane fusion on the surface of the cell at neutral pH, facilitating the entry and spread of NDV. The HN protein plays a significant role in infection by facilitating virus attachment to host cells via sialic acid receptors. These proteins regulate the virulence of NDV and are considered immunogens for the development of any potent vaccine against NDV (23).

The current study is an attempt to express F and HN genes in maize under the activation of constitutive and seed-specific promoters for the production of plant-based vaccines against NDV. The F and HN proteins accumulate in maize kernels, and feeding chickens kernels that contain the target protein should induce the production of antibodies that generate immunity against NDV infection in chickens. Seeds are preferable to green tissues for analyzing protein concentration because seeds are more easily purified, exhibit long-term stability, and accumulate more protein (24, 25).

## Methodology

### NDV Strain Cultivation

A virulent avian avulavirus 1 strain chicken/SPVC/Karachi of Newcastle disease virus originally derived from vaccine strain Mukteswar Mesogenic strain (GU182327 was obtained from the Veterinary Research Institute Lahore, Pakistan. It was manipulated in a special containment facility at CEMB Lahore, Pakistan. Embryonated serum pathogen-free (SPF) chicken eggs, were inoculated with diluted virus (10^5^-10^6^ PFU/ ml phosphate buffer saline (PBS). After incubation at 37°C for 48 h, the eggs were chilled at 4°C to avoid blood contamination. Allantoic fluid samples were harvested and proceeded for viral RNA extraction.

### Complementary DNA (cDNA) Synthesis

Viral RNA was extracted by TRIzol direct RNA extraction method as described by Chomczynski and Sacchi (26). RNA was quantified through Nanodrop (Thermo Scientific). RevertAid first-strand cDNA synthesis kit (Thermo Scientific, K1622) was used for one-step RT-PCR to synthesize cDNA with random hexamers primers. cDNA was prepared in a thermocycler in a single step reaction by placing it to 25°C for 10 min, proceeded by 42°C for 60 min and, 70°C for 5 min. The cDNA was stored at −20°C.

### PCR Amplification of F and HN Genes From cDNA

The NDV cDNA was used as a template to amplify 1,662 bp F gene by using gene-specific [F-Forward 5′CCAGTACCTCTAATGCTGACCATAC3′ and F-Reverse 5′TCACATTTTTGTAACAGCTCTCATCT3′] and 1,712 bp to amplify HN gene by using gene- specific [HN-Forward 5′GACAGCGCAGTTAGCCAAGTT3′ and HN-Reverse 5′TTAAACCCCACCATCCTTGAG3′] as upper and lower primers, respectively. Primers were designed using sequences available at NCBI (accession #GU182327). PCR conditions were set to be 94°C for 5 min, 30 cycles of 94°C for 1 min, 61°C for 1 min, 72°C for 1 min, and 72°C for 10 min.

### TA Cloning and Transformation of F and HN Genes

The amplified PCR product of F and HN genes (of ~1,662 bp and ~1,712 bp, respectively) was resolved on 0.8% agarose gel. The bands were excised with a sharp surgical blade under UV light using safety glasses. PCR products were eluted from gel slices having F and HN genes using a GeneJet™ Gel Extraction kit (Thermo Scientific Cat#k0692). The eluted PCR product of F and HN gene was directly cloned in TA cloning vector as per the manufacturer's protocol (Invitrogen, Cat # K 4500-01). The resultant plasmid was transformed into *E. coli* (Top 10) competent cells for maintenance and propagation purposes. Positive clones of both genes (F and HN) were selected via blue/white screening. Lysogeny Broth (LB) containing kanamycin (50 μg/ml) and tetracycline (12.5 μg/ml) were used to grow bacteria. PCR analysis (with the same primers as mentioned above) was performed for confirmation of positive clones. Restriction digestion was also done to determine the compactness of PCR confirmed TA ligates with EcoR1. Positive clones of both (F and HN) genes were further confirmed through Sanger's sequencing method.

### Cloning, Expression, and Purification of F and HN Protein

The expression of F and HN gene was initially verified in *E. coli* by SDS PAGE, followed by immunoblot detection using polyclonal anti-F antibodies and monoclonal anti-HN antibodies 200 μg/ml: SantaCruze). The F and HN genes were cloned individually with EcoR1 site into the pET-30a vector (obtained from CEMB, Lahore). For the expression of both F and HN genes in *E. coli*, the plasmids were transformed into DE3 (BL-21) cells. Cells were induced at 0.6–0.8 O.D._600_ with IPTG and incubated on a rotary shaker at 30°C for 16 h. The F and HN protein isolated from *E. coli* were purified with Ni2^+^-NTA affinity chromatography under denaturing conditions. Purified protein samples were resolved on SDA PAGE and confirmed through western blot analysis. Quantification of F and HN purified protein was done through ELISA by using gene- specific (polyclonal anti F antibody raised in rabbit) and monoclonal HN antibody (200 μg/ml: Santacruz).

### Plasmid Construction for Plant Transformation

Standard molecular cloning techniques were used for the construction of expression vector to be used for plant transformation. The F and HN genes were amplified with primers having NCO1 sequence at 5' and 3' ends for F gene while Xba1 at 5' and BamH1 at 3' ends of primers for HN which introduced NCO1 sites at 5' and 3' of F gene and Xba1 at 5' and BamH1 at 3' of the HN gene. The synthetic zein promoter was cloned at Sal1 site at 5' and Xba1 site at 3' while Nos terminator was cloned at BamH1 site at 5' and Kpn1 site at 3'. Binary vector pCAMBIA 1301 carrying both CaMV35S promoter, Zea mays zein specific promoter and Nos terminator was used for the construction of pCAMBIA F+HN plasmid. The F gene was cloned under the constitutive promoter while HN gene was cloned under the regulation of (Zein) the seed- specific promoter. The resultant plasmid was verified by PCR and restriction digestion. One of the positive clones was transformed in *Agrobacterium* strain LBA4404 via electroporation following standard protocols.

### Maize (Zea Mays) Transformation

Ears containing the immature embryos were harvested from the inbred lines planted in CEMB greenhouse. The shoot cut method of plant transformation with some modifications already established at CEMB was used in this study. The isolated immature embryos were cut at specific points with a sharp blade under a light microscope. The embryos were transferred in overnight grown *Agrobacterium* suspension of the F+HN gene for co-cultivation and kept at shaker for 1 h at 28°C. The suspension was later discarded and embryos after drying on autoclaved filter paper were shifted on fresh MS plates with auxin and kinetin supplements. The plates were kept at 25°C in the growth room after co -cultivation. After 3–4 days emerging plants were shifted in MS tubes supplemented with kanamycin (50 μg/ml), rooting and shooting hormones, kinetin (1 mg/ml), cytokinin (1 mg/ml) and B5 vitamin complex. The transformation efficiency of the plants was calculated every 10 days depending upon their survival on selection media. Soil mixture with equal quantities of clay and sand was used for the transfer of plantlets to pots. After proper acclimatization, the plants were finally shifted to greenhouse.

### Molecular Analysis

#### Polymerase Chain Reaction

Genomic DNA was isolated from leaves and grains of control and transgenic maize plants by following the protocol as described by Saha et al. (27). PCR analysis was performed by using the gene -specific primers. For the F gene amplification of 587 bp [forward 5'- GAGCGACTTGGACCTGTATTG−3' and reverse 3'- GCGTCTTTTGTTGTGCCTTT−5'] and HN gene amplification of 567 bp [Forward 5'- ACTATCCTGGTGTCGGTGGT-3' and reverse 3'- CGCCCCTAAGTGTATGGTTC−5'] primers were used. Amplification was done at 94°C for 5 min, 94°C for 1 min, 61°C for 1 min, 72°C for 1 min followed by 30 cycles and 72°C for 10 min. The PCR product was analyzed on 0.8% gel and visualized under UV light to confirm the presence of F and HN genes.

### Expression Studies of Transgenic Maize Plants

Total RNA was isolated from leaves and seeds of transgenic maize plants following the method described by Hou et al. (28) followed by synthesis of cDNA using a cDNA synthesis kit (Thermo Scientific, K1622). The mRNA expression of F and HN was done through Piko- Real-Time PCR Thermo scientific by using Maxima SYBR Green/ROX qPCR Master Mix (2X) (Thermo Scientific, K0221). As an internal control housekeeping gene GAPDH (Glyceraldehyde 3-phosphate dehydrogenase) was used for data normalization. Two hundred nanogram of cDNA was used as a template in RT- PCR. Gene- specific primers [Forward primer 5'AAGCACAACCGAAGGATTTG 3' Reverse primer 3'GCCGCTCAAACAGGAATAAA 5'] and [Forward Primer 5'ATATCCCGCAGTCGCATAAC3' Reverse primer 5'TTAAACCCCACCATCCTTGA3'] were used to amplify 191 and 172 bp of F and HN gene, respectively, for comparative quantification through RT-PCR. The cycling conditions were programmed with initial denaturation of 4 min at 96°C, followed by 35 cycles of amplification (96°C for 30 s, 59°C for 45 s, and 72°C for 45 s). Statistical analysis was used to observe CT values obtained from different transgenic maize plants normalized with housekeeping GAPDH relative control.

### Protein Analysis

#### Estimation of F and HN Protein Through ELISA

Total soluble protein was extracted, from 200 mg corn leaves and seeds by grounding in liquid nitrogen and resuspending the powder in 400 μl of freshly prepared ice-cold plant extraction buffer PBST comprising of [100 mM NaCl, 10 mM Na_2_ HPO_4_, 3 mM KH_2_ PO _4_(pH 7.2), and 0.05% Tween-20 (v/v)]. Samples were subjected to centrifugation with a speed of 13,000 g for 15 min at 4°C after homogenization on ice for 5 min. Protein concentration in each sample was determined by using the Bradford assay (29). ELISA and western blot analysis were performed according to the protocol described by Shil et al. (30) and Su et al. (31). For ELISA plates were coated with 100 μl of gene- specific (polyclonal anti F antibody raised in rabbit) and monoclonal HN antibody (200 μg/ml: Santacruze) diluted in PBST 4°C for 24 h. Antibody coated ELISA plates were washed with PBST (100 μl). Blocking was done with PBST having 3% skim milk powder and incubated at room temperature for 2 h. Crude protein extracts from leaves and seeds of transgenic corn were added to ELISA plates and left for 2 h at 37°C. Plates were washed again with PBST. For detection of F and HN proteins, 100 μl of secondary antibody anti-chick IgY (2.5:10000) and anti-mouse with (1:10000) diluted in PBST was added in each well by keeping at 37°C followed by application of substrate BCP/NBIT followed by incubation of half an hour at 37°C for color development. HCL (1N) was added to stop the reaction. Optical density was calculated at 450 nm and values were compared with the standard curve for quantification of F and HN proteins. The Given formula was used for calculation of total soluble F and HN proteins.

**% of TSP=TP/TSPx100**

TP= protein concentration obtained from ELISA

TSP = protein concentration obtained from Bradford assay.

#### Detection of Total Soluble Protein Through Western Blot Analysis

Freshly extracted proteins (100 μg) from both seeds and leaves of transgenic maize plants were resolved on 12% SDS PAGE (32). The Gel initially proceeded at 85 volts until it was transferred to resolving gel than the voltage was increased upto a total of 110 volts. For the western blot analysis, the isolated protein was transferred to a nitrocellulose membrane by using trans blotter (Mini transblot cell BIORAD). NDV specific primary antibody and anti-chick IgY secondary antibody for F while monoclonal HN primary antibody and anti-mouse secondary antibody for HN protein were used, respectively, for antibody -antigen reaction to confirm the specificity of both the proteins.

#### Immunization of Chicks for Immune Response

The transgenic maize plants with increased expression of F and HN genes were raised to their advanced generation for their confirmation of immunogenic response against NDV in chicks fed with transgenic maize seed. All procedures of handling the animals were performed following the ethical standards of the institution and the study was approved by the ethical committee of the institution (CEMB), University of the Punjab, Lahore Pakistan (Ref, CEMB/IAEC/18-10; August 2019). Broiler chickens not receiving NDV vaccination (aged 15 days) were taken from the poultry industry (poultry farm) and kept in animal house conditions to be acclimatized for 3 days before subjected to immunogenic response studies planned at CEMB. The animals were kept in groups based on deep litter system of animal management system with optimum temperature of 23°C±_3°C, relative humidity 50 to 60% and 12 h light and 12 h dark period. Thus, all chickens were of the same age (i.e., 15 days old). After 3 days acclimitization, then chicken in all groups were immunized exactly at aged 18 days old. The chickens were divided into 5 groups with each group having 5 chickens. Group 1 was named as a control group and it was given the non-transgenic maize diet. Group 2 chickens were fed with transgenic corn leaves and seeds comprising of 200 μg F and HN proteins at specific intervals of 0, 10, 20, 30, 40, 50, and 60 days. All the chickens were kept deprived of food for 4 to 5 h before immunization and 1 to 2 h after immunization. The third group was injected with a 20 μg of recombinant F protein from *E. coil* and the fourth group was immunized with 20 μg of recombinant HN protein from *E. coli*, respectively, at specific intervals of 0, 10, 20, 30, 40, 50, and 60 days. Similarly, the fifth group was vaccinated with the recommended dose of commercially available Lasota live vaccine against NDV orally through drinking water immediately after acclimitization (18 days old). Moreover, all the chickens of groups 2 (receiving transgenic maize), 3 (receiving *E.coli* produced F protein), and 4 (receiving E.coli produced HN protein) were subjected to priming with 0.2 mg/ml of complete Freund's adjuvant (CFA;) and booster dose of incomplete Frerund's adjuvant after 15 days interval by a subcutaneous injection. All chicks were fed *ad libitum*.

#### Preparation of Serum Samples

The blood was collected after each immunization at specific intervals of (0, 10, 20, 30, 40, 50, and 60 days) followed by incubation at 25°C for 45 min. After the incubation the serum was collected from blood samples through centrifugation (5,000 × g) at 4°C for 15 min. The sera collected at different times intervals were preserved at −20°C until proceeding for further experiments.

#### Estimation of Serum IgY Response in Immunized Chickens

Serum specific F and HN antibodies were determined through ELISA as mention in protocols by Habibi et al. (33) and Xiao and Daniell (34). In order to confirm the presence of F and HN specific antibodies in the sera of immunized animals, briefly; a microtiter plate was coated with 0.5 ug of purified F and HN proteins diluted in PBS, kept at 4°C for 24 h. Next, the previously obtained serum was added in each well for IgY antibody determination and incubated for an hour. The serum samples were used in 1: 40 dilutions. Following 1 h incubation, the commercial HRP conjugated goat to anti-chick IgY (ab 6877) was added as a secondary antibody in each well. After blocking with 1N HCL OD values were calculated at 450 nm to find out IgY levels in serum samples.

#### Determination of Follicle- Stimulating and Luteinization Hormones

To further determined the impact of plant -derived orally fed F and HN protein on chicks health, the activity of sex -related hormones (Follicle stimulating hormone, Luteinizing hormone) in the serum of broilers was also evaluated in all three groups (G1;G2;G5) at five different experimental stages (0, 10, 20, 30, 40, 50, and 60) through ELISA assay, using commercially available ELISA kit as per manufacturer protocol (Chicken LH ELISA kit MBS 008505, Chicken FSH ELISA kit MBS 260290).

### Statistical Analysis

All data were analyzed using analysis of variance in a statistical analysis program (GraphPad Prism version 7) for Windows. To determine significant differences, the means of all treatments were compared using Tukey's multiple comparison test. Differences were considered significant when P < 0.05. GraphPad Prism software was used to perform statistical analysis.

## Results

### Evaluation of (F and HN/pCAMBIA and PET) Clones

**Figure 1:**
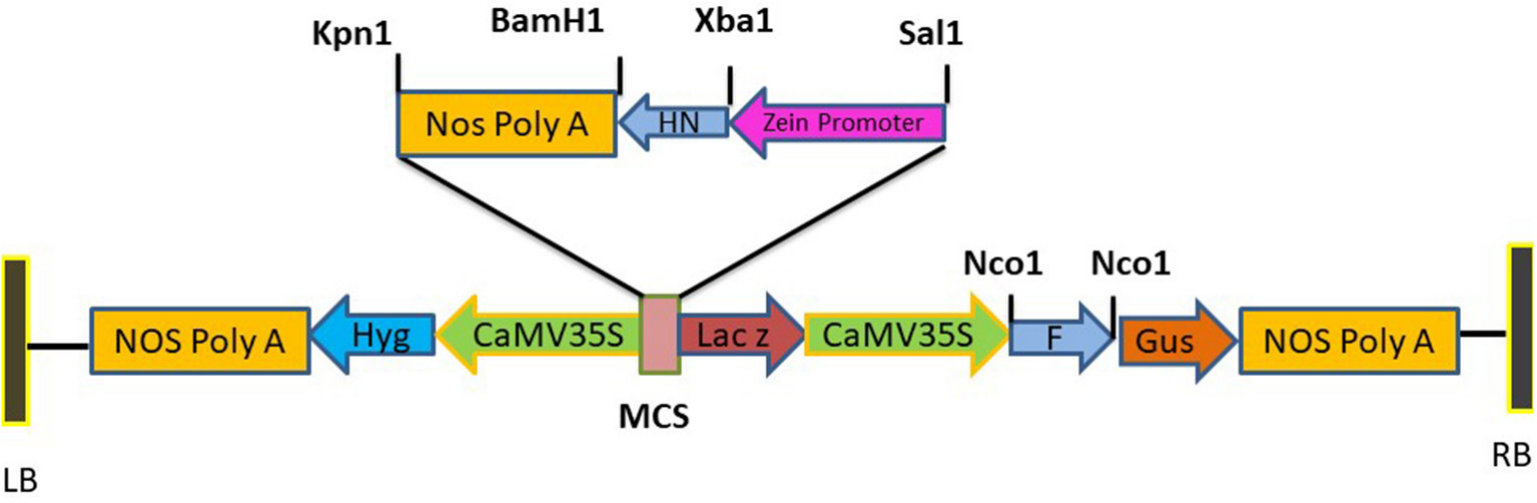
Diagrammatic illustration of recombinant plasmid pCAMBIA F+HN. The immunogenic F gene was cloned in Nco1 and Bgl11 site under the regulation Cauliflower mosaic virus (CaMV) 35S promoter and HN gene was cloned in Sal1 and Kpn1 site of multiple cloning site with seed specific promoter.

**Figure 2:**
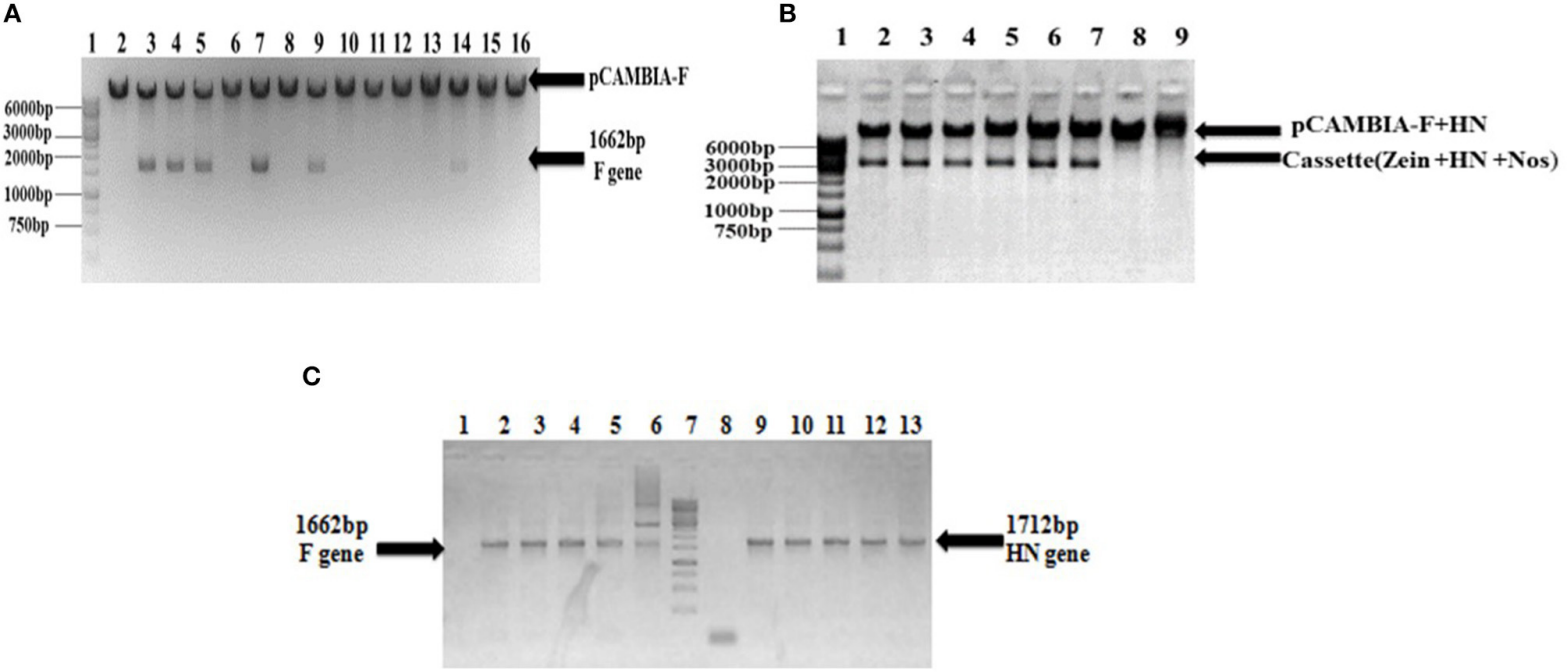
Confirmation of recombinant pCAMBIA F+HN through PCR and restriction digestion. **(A)** Recombinant plasmid was digested with Nco1 and Bgl11 to confirm the presence of F gene and excised fragment of 1,662 bp confirm the presence of F gene. **(B)** The whole cassette comprised of HN gene, zein specific promoter and Nos terminator was digested with Sal1 and Kpn1 and excised fragment at 3,366 bp confirmed the presence of whole cassette along with HN gene in recombinant plasmid. **(C)** PCR analysis of transformed clones. PCR carried with full length F and HN gene specific primers. Lanes 3–6, full-length F gene primer led to 1,662 bp amplified product; Lane 1, shows amplification from non-transformed clones and Lane 2, positive control. Lane 9–12, the full length HN gene primer led to 1,712 bp amplified product; Lane 8, showing amplification from non-transformed clones; Lane 13, positive control.

**Figure 3:**
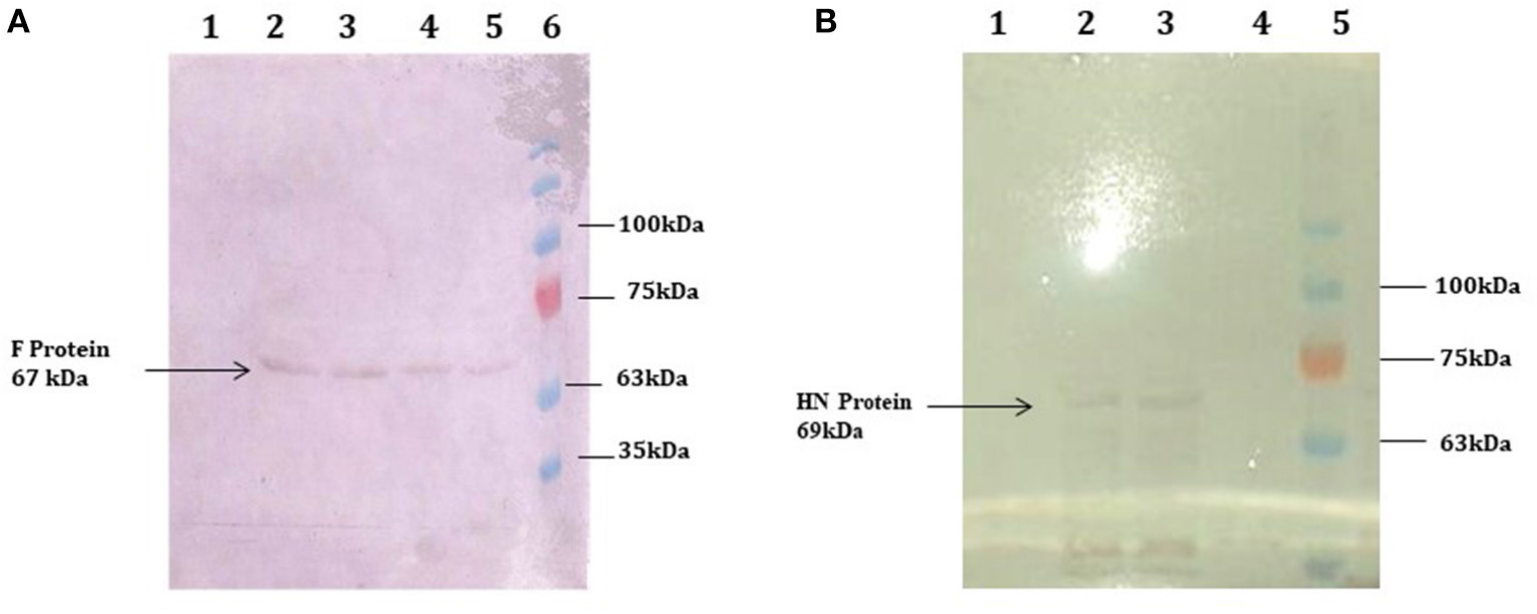
Western blot analysis demonstrating the expression of F and HN protein *E. coli*. **(A)** purified protein through ion exchange chromatography extracted from transformed Rosetta cells. A 67 kDa protein band obtained through western blot analysis confirm the expression of F protein. Lane 1, Negative control (Untransformed Rosetta cells); Lanes 2–5, F-transformed in Rosetta cells; Lane 6, showing Pre-stained protein marker (Fermentas) **(B)** purified protein through ion exchange chromatography extracted from transformed Rosetta cells. A 69 kDa protein band obtained through western blot analysis confirm the expression of HN protein. Lane 1, Negative control (Untransformed Rosetta cells); Lanes 2 and 3, HN-Transformed in Rosetta cells; lane 5, Pre-stained Protein marker (Fermentas).

The full-length F (~1,662 bp) and HN (~1,712 bp) genes were successfully amplified after reverse transcription of extracted viral RNA from a virulent strain of the virus. The F gene with NCOI restriction site under the regulation of ubiquitin promoter while HN gene with Xba1 and BamH1 restriction site at the N- and C-terminals, respectively, under the control of seed -specific promoter were ligated in pCAMBIA 1301 vector constructing recombinant p. CAM F-HN plasmid (Figure 1). The amplification of (~1,662 bp) and (~1,712 bp) F and HN genes, respectively, through PCR by using gene -specific primers and elimination of 1,662 bp fragment from 11,837 bp pCAMBIA 1301 vector for F while 1,712 bp for HN confirmed the successful ligation of both gene in the plant expression vector (Figures 2A–C). Further, the appearance of all comparative nucleotide sequences after being sequenced through the sanger method further confirmed the sequence specificity of cloned genes in p. CAMBIA F-HN recombinant plasmid. The full-length F and HN genes were also ligated into the pET 30a vector to produce recombinant pET- F and pET-HN plasmid for its expression in *E. coli*. The obtained fraction of expressed proteins in soluble form observed through SDS depicted a sharp 67 kDA band for F protein and 69 kDA protein for HN protein. The purified F and HN protein resulted in 32 ng/μl and 34.1 ng/μl yield, respectively. The purified protein when subjected to western blot analysis demonstrated a single 67 kDA band for F and 69 kDA for HN protein through (polyclonal anti F antibody raised in rabbit) and monoclonal HN antibody (200 μg/ml: Santacruz) (Figures 3A,B).

### Generation of Transgenic Maize Producing NDV Antigens

**Figure 4:**
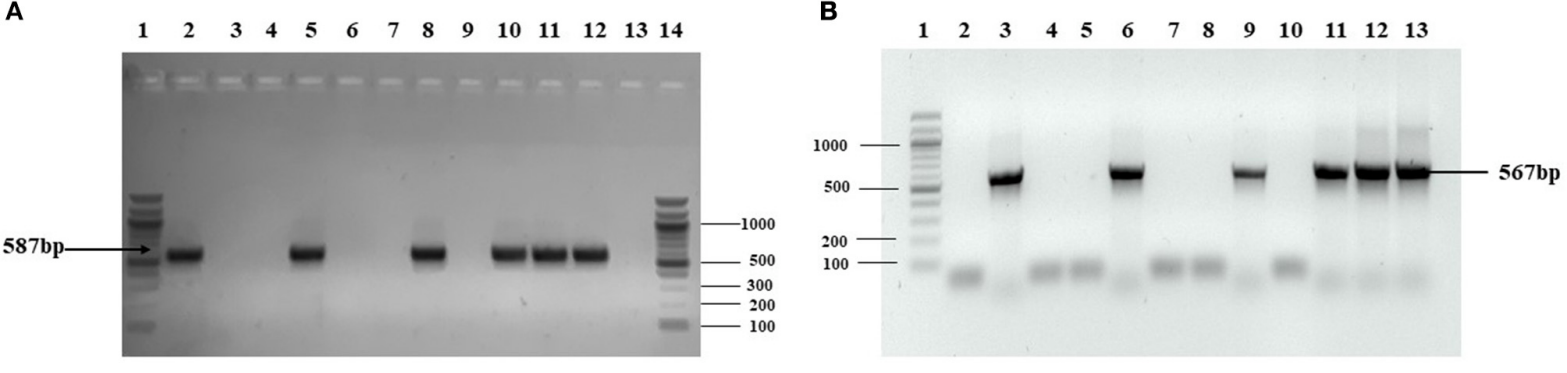
PCR analysis of putative transgenic maize plants. Genomic DNA extracted from transgenic leaves and corn seeds was used as template for amplification. Amplification from putative maize plants transformed by agrobacterium mediated transformation. **(A)** PCR was carried with gene specific for F gene and amplified 587 bp product size confirmed the presence of F gene in five plants out of ten transformed plants (P1–P10). Lane 1 is 100 bp ladder; Lane 2, positive control; Lane 3–12, PCR amplification from transformed plants (P1–P10) in which P3, P6, P8, P9, and P10 confirmed the presence of F gene in maize leaves; Lane 13, PCR amplification from non-transgenic plant; Lane 14, 100 bp ladder. **(B)** PCR was carried with gene specific for HN gene and amplified 567 bp product size confirmed the presence of HN gene in five plants out of 10 transformed plants. Lane 1, 1 kb ladder; Lane 2, the PCR from non-transgenic plant; Lane 3, positive control; Lane 4–13, PCR amplification from transformed plants (P1–P10) in which P3, P6, P8, P9, and P10 confirmed the presence of HN gene in maize seeds.

**Figure 5:**
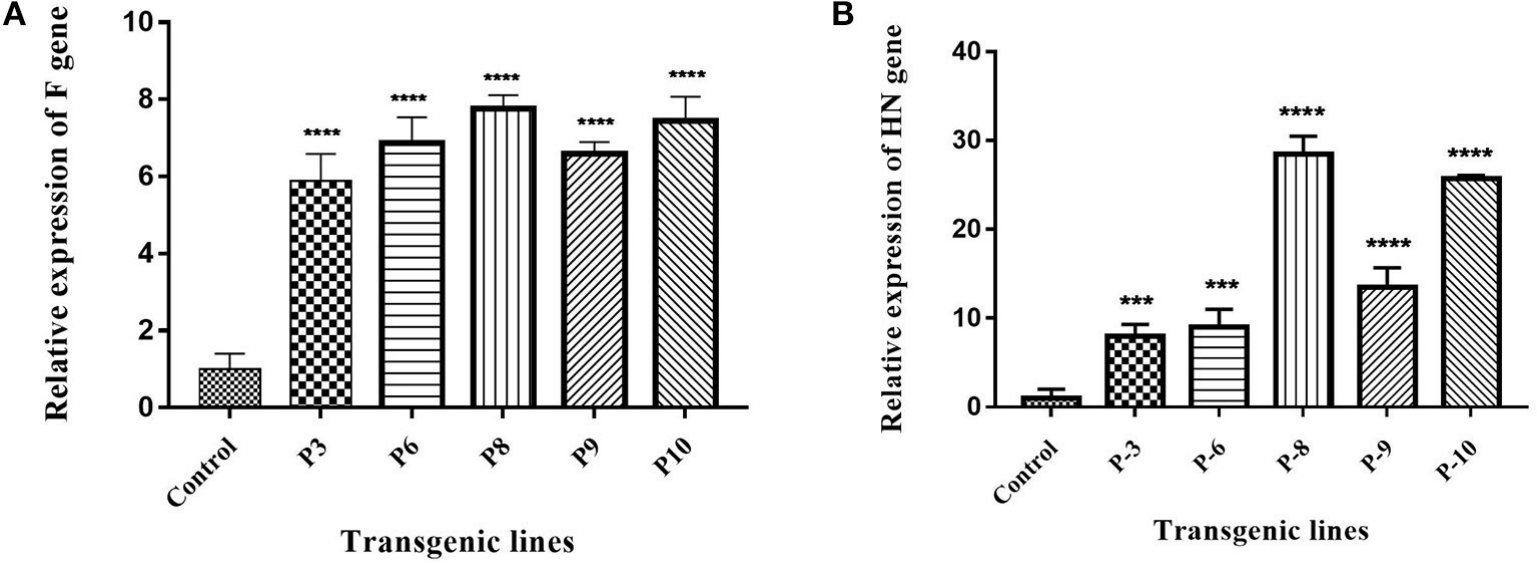
Expression analysis of F and HN gene through Real Time PCR. **(A)** cDNA was synthesized from transgenic plants and expression of F gene was studied at transcription level. The maximum expression of 7-fold in P8 and P10 was observed as compared to 5-fold in P3, 6-fold in P6 and P9. **(B)** cDNA was synthesized from transgenic plants and expression of HN gene was studied at transcription level. 28-fold expression of HN mRNA in P8, 25-fold in P10, 13-fold in P9 was obtained as compared to P3, P6 where it remained 8–9-fold. Data shown are average ± SD of three biological replicates. The significance of the data is determined by one-way ANOVA and *p* < 0.0001 is indicated by “****” and *p* < 0.001 is indicated by “***” above bars.

**Figure 6:**
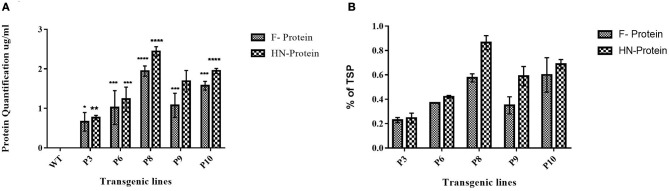
Quantification of F and HN protein in transgenic maize leaves and seeds. Quantification of F and HN protein was done through ELISA. Percent of TSP was calculated by comparing the absorbance to the Standard plot of purified F and HN through ELISA. **(A)** The concentration of F protein in P6 and P8 showed highest protein concentration of 1.5 and 1.66 μg/ml in leaf, respectively. While the highest concentration of HN protein 2.4 and 1.95 μg/ml of total seeds protein was calculated in P8 and P9, respectively. Data shown are average ± SD of three biological replicates. The significance of the data is determined by one-way ANOVA and *p* < 0.0001 is indicated by “****,” *p* < 0.001 is indicated by “***,” *p* < 0.01 is indicated by “**” and *p* = 0.01 is indicated by “*” above bars **(B)** Similarly, the highest TSP was observed in P8 for both F and HN which was 0.7% and % of 0.8%, respectively. Data shown are average ± SD of three biological replicates.

**Figure 7:**
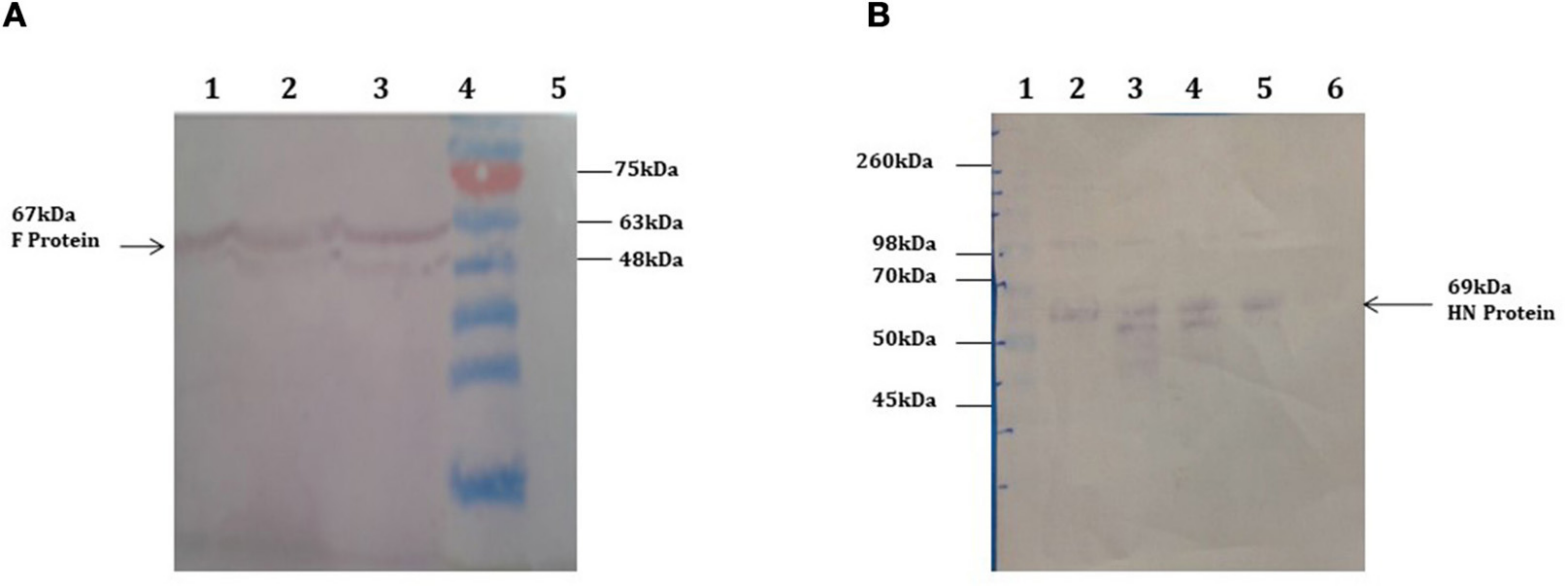
Western blot analysis demonstrating the expression of F and HN protein in transgenic plants. Total soluble protein extracted from transgenic maize leaves and seeds transformed by agrobacterium mediated transformation. **(A)** 67 kDa protein band was obtained by western blot from transgenic maize leaves confirms the expression of F protein in leaves. Lanes 1–3, TSP from transgenic plants showing 67 kda F protein; Lane 4, Pre-stained protein marker (Fermentas); Lane 5, Negative control (wild type plant). **(B)** 69 kDa protein band was obtained by western blot from transgenic maize seeds confirms the expression of HN protein in seeds. Lane 1, protein marker Lanes 2–5, TSP from transgenic seeds showing 69 kda HN protein; Lane 6, Negative control (wild type plant).

*Agrobacterium*-mediated nuclear transformation was carried out to transform inbred lines of maize with NDV F and HN genes under the regulation of seed -specific and constitutive promoters. After transformation, 10 transgenic maize lines bearing F and HN gene were Produced. The presence of recombinant plasmid p CAMBIA F-HN in transgenic maize plants was confirmed through amplification of the F and HN gene by using gene- specific primers. The appearance of amplified products at a specific size of 587 and 567 bp for F and HN genes, respectively, in five transgenic maize plants namely P3, P6, P8, P9, and P10 confirmed the successful introduction of F and HN gene in maize (Figures 4A,B). Reverse transcription of F and HN genes RNA isolated from transgenic maize plants namely P3, P6, P8, P9, P10 along with non-transgenic control maize plant subjected to the amplification by using gene -specific primers resulted in 587 bp for F and 567 bp for HN gene The mRNA expression of F gene through real- time PCR determined 7-fold expression in P8 as compared to 5-fold in P3, 6-fold in P6, P9 and 7-fold in P10 (Figure 5A). Interestingly 28-fold expression of HN mRNA in P8, 25-fold in P10, 13-fold in P9 was obtained as compared to P3, P6 where it remained to be 8–9-fold (Figure 5B). The recombinant F and HN proteins in transgenic maize leaves and seeds were determined through direct ELISA. The analysis demonstrated that plants P6 and P8 showed the highest F protein concentration of 1.5 μg/ml and 1.66 μg/ml in leaf, respectively (Figure 6A). While the highest concentration of HN protein 2.4 μg/ml and 1.95 μg/ml of total seeds protein was calculated in P8 and P9, respectively (Figure 6A). The concentration of TSP was calculated for both F and HN protein by using the formula mentioned in section Estimation of F and HN Protein Through ELISA. It was further illustrated that both F and HN proteins were calculated to be in the range of 0.5 and 0.8% of TSP with highest in P8 for both F and HN which was 0.7 and 0.8% of TSP, respectively (Figure 6B). Further, the appearance of 67 and 69 kDa bands after the color reaction of a nitrocellulose membrane confirmed the specificity of protein analyzed (Figures 7A,B).

### Comparative Study of Serum IgY Immune Responses

**Figure 8:**
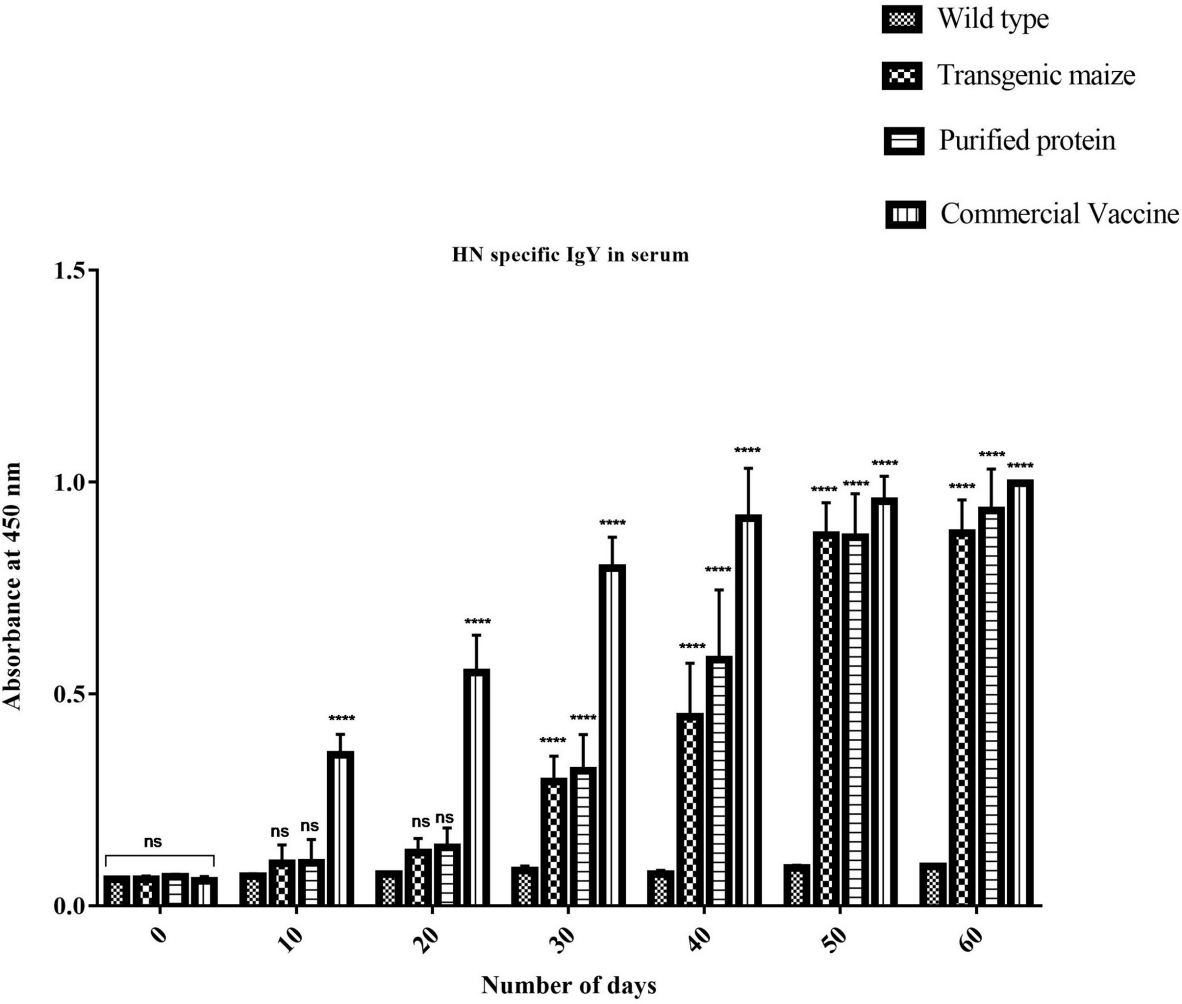
Serum specific IgY response against HN protein. HN specific Serum IgY immune responses in Chicken immunized with transgenic maize seeds. Groups of 18 days old chicken were immunized on day 0, 10, 20, 30, 40, 50, and 60. IgY level against HN protein was almost similar at 50 and 60 days immunization to the chicken vaccinated through injection of recombinant HN protein from *E. coli* and commercially available vaccine against NDV. While no immunogenic response was observed in group receiving non-transgenic maize. Data shown are average ± SD (*n* = 5/group). The significance of the data is determined by one-way ANOVA and *p* < 0.0001 is indicated by “****” above bars whereas “ns,” non-significant.

**Figure 9:**
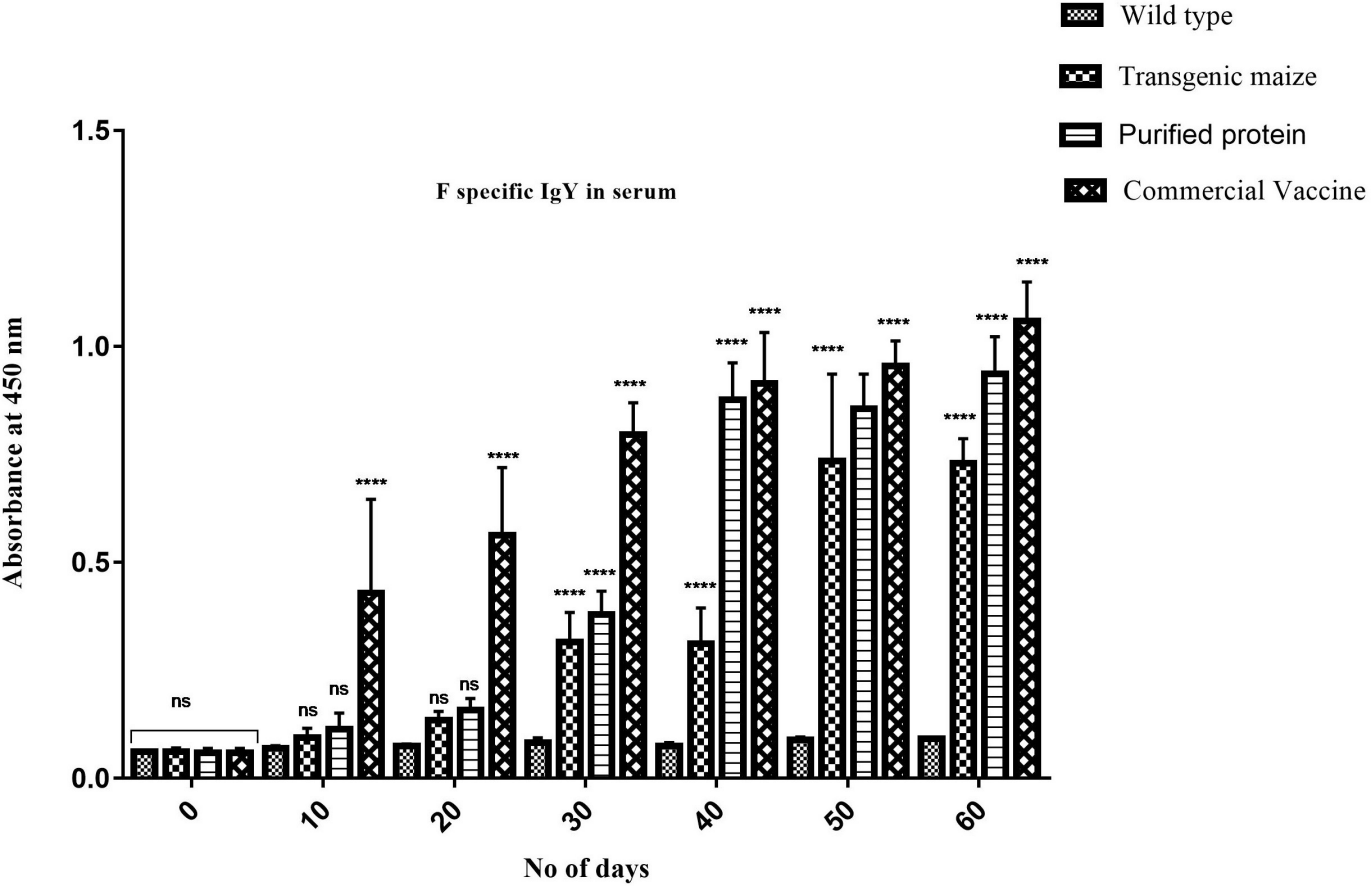
Serum specific IgY response against F protein. F specific Serum IgY immune responses in Chicken immunized with transgenic maize leaves. Groups of 18 days old chicken were immunized on day 0, 10, 20, 30, 40, 50, and 60. IgY level against F protein was slightly less as compared to the to the chicken vaccinated through injection of recombinant F protein from *E. coli* and commercially available vaccine against NDV while no immunogenic response was observed in group receiving non-transgenic corn. Data shown are average ± SD (*n* = 5/group). The significance of the data is determined by one-way ANOVA and *p* < 0.0001 is indicated by “****” above bars whereas “ns,” non-significant.

Maize leaves and seeds from transgenic plants were fed to chickens three times a week for 60 days to determine the induced serum immune response against NDV in comparison to chicken fed with a normal diet. Immunized chickens were bled after every 10 days and NDV specific antibodies against F and HN proteins were measured using ELISA at different time intervals. The immunized chickens receiving transgenic corn showed a strong immunogenic response. The serum IgY level against HN protein was almost similar at 50 and 60 days immunized chicken in groups vaccinated through injection of recombinant HN protein from *E. coli* and the commercially available vaccine against NDV as shown in **Figure 9** but the serum IgY level against F protein was slightly less in chicken vaccinated through injection of recombinant F protein from *E. coli* as compared to commercially available vaccine against NDV while no immunogenic response was observed in the group receiving non-transgenic corn (Figure 8). This may be due to less concentration of total soluble F protein or usage of seed- specific promoter with HN protein gene as compared to constitutive with F protein gene. The immunogenic response developed in chicken after first feeding was enhanced with each booster dose exactly similar to that of commercial vaccine treated chicks. The highest antibodies level was calculated after 50 days which persist at the same level up to 60 days as compared to the chickens receiving non-transgenic maize showing no immunogenic response (Figure 9).

### Evaluation of Sex-Related Hormonal Response

**Figure 10:**
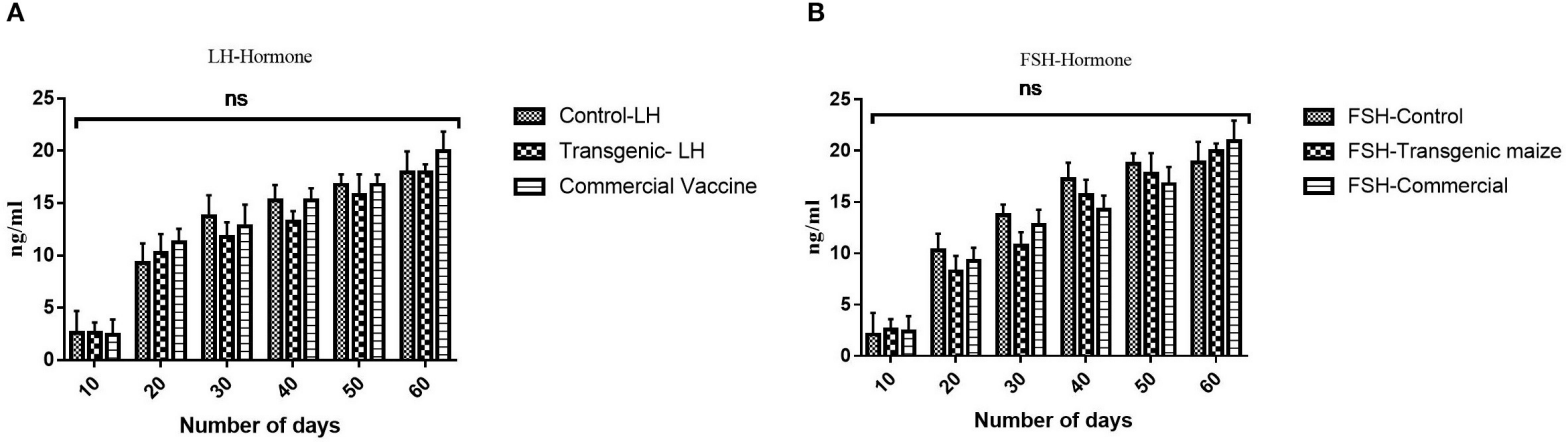
Determination of Sex Hormones. **(A)** The level of luteinizing hormone in three groups receiving transgenic diet; non-transgenic maize and commercially available vaccines immunized at different time interval (0, 10, 20, 30, 40, 50) were also evaluated to check any disturbance in Luteinizing hormone. No statistically significant difference was observed between three treated groups **(B)** The level of follicle stimulating hormone in three groups receiving transgenic diet; non-transgenic maize and commercially available vaccines immunized at different time interval (0, 10, 20, 30, 40, 50) were also evaluated to check any disturbance in follicle stimulating hormone. Data shown are average ± SD (*n* = 5/group). “ns,” non-significant difference was observed between three treated groups.

Immunized chicken with all groups receiving transgenic maize, wild type, commercial vaccines and *E. coli* produced were also examined for sex hormones determination to determine the impact of orally fed proteins on fertility of chickens receiving genetically modified corn. No statistically significant difference was observed between three treated groups (Figures 10A,B).

## Discussion

The delivery of recombinant proteins as vaccine after inducing expression in plants is an attractive alternative to injectables, especially in developing countries. The oral administration of plant-derived antigens against different diseases has previously been reported by many authors (6, 15, 35–39). The immunogenic response generated against many orally administered proteins of pathogens after expression in transgenic plants (8, 33) has encouraged study on the expression of other antigens in plants, with defined goals of edible vaccine development (40). We demonstrate the cloning, expression, and immunogenicity of the F and HN immunodominant glycoproteins from NDV in transgenic maize plants, as previously described (17, 41–43).

The expression of the F gene under the constitutive CaMV 35S promoter and the HN gene under a seed-specific promoter was designed. The results indicated that both genes were successfully expressed in maize plants. After developing plantlets from Agrobacterium-inoculated embryos of maize, isolated genomic DNA was screened for successful introduction of both F and HN genes by PCR. The amplification of 567 bp for the F gene and 587 bp for the HN gene obtained in 5 out of 10 maize plants tested through PCR using gene-specific primers confirmed the presence of F and HN genes in transformed maize plants. The successfully amplified plants were evaluated through mRNA expression as previously described (42).

mRNA expression of the F gene, determined by real-time PCR, indicated a 7-fold increase in P8, 5-fold in P3, 6-fold in P6 and P9, and 7-fold in P10. There expression of HN mRNA increased 28-fold in P8, 25-fold in P10, and 13-fold in P9, compared to that in P3 and P6 of 8–9-fold). Additionally, the F and HN proteins expressed in transgenic maize plants were detected and quantified using anti-NDV antisera in ELSIA (38). The levels of expression of F and HN proteins in both leaves and seeds were in accordance with protein levels reported by other researchers (23, 42). The orally fed, plant-derived NDV protein was able to induce a specific immune response in chickens compared to injected *E. coli*-expressed F and HN proteins and a commercially available NDV vaccine. The chickens orally fed a transgenic maize diet showed a specific anti-NDV response in analyzed sera, clearly demonstrating that transgenic maize plants were able to express F and HN proteins, resulting in the production of anti-NDV antibodies in chickens. The identification of IgY antibodies in the sera of chickens that had been orally fed transgenic maize further indicated that plant-based vaccines were able to induce both systemic and mucosal immunity. The levels of IgY in orally fed chickens were comparable to those of chickens injected with *E. coli*-expressed immunogenic protein and a commercially available NDV vaccine. Local IgY production is an important part of mucosal immune response comparable to traditional mucosal immunization as demonstrated by various studies (44, 45).

These results are in agreement with those of a study that showed immunized mice fed eHN protein from tobacco cells exhibited a gradual increase in immune response in the presence of incomplete adjuvant (46). The induced immune response in immunized chicks demonstrated that transformed genes produced active proteins as stated by Lai et al. (46) and Gorantala et al. (47). An induced immune response has also been reported in immunized chicks fed transgenic maize expressing F protein (41). Furthermore, the increase in immune response was greater against the HN protein as compared to the F protein. This is suspected to be due to the use of a seed-specific promoter for increased expression of the HN gene. Similarly, previous research demonstrated that transformed tomatoes with the CTB gene under the regulation of the CaMV 35S promoter accumulated 0.2 to 0.4% TSP as compared to the same transformed tomato plants with the CTB gene under control of the E8 promoter (tomato fruit-specific promoter), which exhibited significantly enhanced expression of ~0.8% TSP (48). The oral diet experiments in the current study were carried out on chickens to determine the efficacy and feasibility of using transgenic maize as an edible vaccine in poultry. The effectiveness, economic benefit, ease of administration, and ability to provide mucosal immunity to avian species will lead to further application of orally fed immunogens (49).

## Conclusion

The study demonstarte that NDV antigens produced in tramsgemic maize are able to induce immune response in chicken fed orally with diet and further the immune response generated is of mucosal and systemic type and is a step forward toward development of edible vaccine in maize.

## Data Availability Statement

All datasets presented in this study are included in the article/supplementary material.

## Ethics Statement

The animal study was reviewed and approved by the ethical committee of the Centre of Excellence in Molecular Biology, University of the Punjab Lahore, Pakistan.

## Author Contributions

NS designed and completed the experiment. TS and SS assisted in experiment. AL helped in experiment designing. AY helped in manuscript write up and experimentation. SA helped in experiment. AS and TH supervised the experiment. AR overall designed the experiment, supervised, and manuscript write up. All authors contributed to the article and approved the submitted version.

## Conflict of Interest

The authors declare that the research was conducted in the absence of any commercial or financial relationships that could be construed as a potential conflict of interest.
